# Socioeconomic status and the relationship of physical performance with activities of daily living among US older adults

**DOI:** 10.1371/journal.pone.0347918

**Published:** 2026-05-20

**Authors:** Grace Chang, Rahul Malhotra, Benjamin Seligman

**Affiliations:** 1 Fielding School of Public Health, University of California, Los Angeles, United States of America; 2 Centre for Ageing Research & Education, Duke-NUS Medical School, Singapore; 3 Health Services Research & Population Health, Duke-NUS Medical School, Singapore; 4 Geriatric Research, Education, and Clinical Center, Veterans Affairs Greater Los Angeles Health Care System, Los Angeles, United States of America; 5 Department of Medicine, David Geffen School of Medicine, University of California, Los Angeles, United States of America; Instituto Nacional de Geriatria, MEXICO

## Abstract

**Background:**

Independence in activities of daily living (ADLs) is an important health outcome for older adults and a predictor of morbidity and mortality. Physical performance is closely associated with ADL independence. It is not clear if socioeconomic status (SES) may alter this relationship so that people with more resources may remain independent at lower performance.

**Objective:**

To assess whether SES is an effect modifier of the association between physical performance and ADL limitations.

**Methods:**

Using data from the Health and Retirement Study, we considered two outcomes in logistic regression models: cross-sectional presence of ADL limitations and longitudinal development of new limitations. We separately considered two performance measures, gait speed (gait) and grip strength (grip), and three SES measures, education, income, and wealth, with and without an interaction term, for a total of 12 regressions. Covariates were age, sex, comorbidity count, cognition, and BMI (for grip) or height (for gait).

**Results:**

Respective sample sizes for gait and grip were 4,825 and 3,401 in cross-sectional analysis and 3,995 and 2,860 longitudinally. They were 57–59% female with median ages 69.0–75.4 years. In regressions without interaction terms, gait and grip were significantly associated with greater odds for the presence or development of ADL limitations (0.95 and 0.97 respectively for grip, 0.03 and 0.20 for gait) while SES measures were not. When interaction terms were included, none reached statistical significance.

**Conclusions:**

We found that SES did not modify the relationship between performance measures and ADL limitations cross-sectionally or longitudinally. Greater SES may not reduce the effort needed for ADLs, or Americans with low SES may similarly access adaptive equipment. Maintaining physical performance remains key for maintaining independence.

## Background

From 2010 to 2020, the population of older adults – people aged 65 years and older – in the U.S. rose by 15.5 million people, the largest proportional rise between any two consecutive decades since 1890 [1]. As the population of older adults in the U.S. grows, more individuals will develop disability [2]. Previous studies illustrate that disability, often associated with functional impairment, is correlated with higher healthcare utilization and costs [3–6]. Disability-related conditions also account for a significant number of disability-adjusted life years (DALYs) and years lived with disability (YLD), population estimates for morbidity and mortality. In the Global Burden of Diseases, Injuries, and Risk Factors Study (GBD) 2019, individuals who qualified for rehabilitation services, programs to improve functional ability, accounted for 310 million YLDs [7]. Other factors undermining function, such as musculoskeletal disorders and falls, also contribute to the significant burden of disability [8,9]. Therefore, understanding what contributes to disability and influences its onset can create opportunities to help older adults maintain their health and well-being.

Disability can be studied through Activities of Daily Living (ADL) limitations, a self-reported measure of functional status to assess one’s ability to care for their basic needs [10]. Assessments of physical function – such as standardized physical performance tests – are strong predictors of self-reported health outcomes like ADL limitations [11–13]. Physical performance tests have been demonstrated to be highly correlated with ADL limitations, as they assess the movements necessary to accomplish these basic tasks [14]. For example, higher grip strength, a marker of muscle strength and physical activity, is strongly associated with less ADL limitations [15,16]. Similarly, higher gait speed, a marker of lower limb strength, mobility, and sensory function, is strongly associated with lower ADL impairment [17,18]. With physical performance tests measuring the physiological and anatomical components to complete ADLs, they are reliable predictors for incident ADL limitations [12,15].

However, ADLs are a self-reported, subjective measure of function, and individuals may perform ADLs differently depending on their environment [19]. For example, disability risk is altered by type of housing, such that risks are greatest among individuals living in poor-quality housing [20]. Socioeconomic status (SES) also plays an important role as a part or determinant of the environment [21]. Generally, higher SES is correlated with better health outcomes, including lower mortality rates [21,22]. Further, SES has been associated with reduced odds of ADL limitations, though the causal mechanism is unclear [23,24]. SES has been shown to influence aspects of an individual’s home environment, such as supportive household equipment including handrails, which may explain the protective nature of higher SES in maximizing one’s functional ability [25,26]. However, it remains unknown older adults of higher SES use their capital to fully overcome physical deficits. Even with the capacity to purchase assistive devices, it is unclear if SES changes the physical threshold necessary to perform the same ADL.

We examine whether SES may be an effect modifier of the relationship between physical performance and ADL limitations in a large, longitudinal cohort of older adults in the U.S., studying both cross-sectional and incident ADL limitations. If higher SES allows individuals to alter their environment and make self-care tasks easier to complete, this may alter the relationship between physical performance and ADL limitations. This may provide a potential mechanism for the association between SES and ADL limitations.

## Methods

### Data source

Data were from the Health and Retirement Study (HRS), a longitudinal cohort representative of U.S. adults aged 50 and above [27,28]. The survey is administered every two years and participants are asked questions including on demographics, health, assets, and income. In every wave, except for 2020, HRS collects data on physical measures during face-to-face interviews from a subset of community-dwelling participants who did not use proxies; individuals are assessed every other wave. We used the 2020 RAND HRS Longitudinal File. Our cross-sectional analysis, using the HRS wave in 2016, was limited to individuals who had either gait speed or grip strength measured in 2016. Participants were excluded for missing data on any SES measure, covariates, or outcome. For our longitudinal analysis, following participants from 2016 to 2018, individuals with 5 ADL limitations in 2016 were excluded to reduce ceiling effects.

All data used were publicly available and de-identified; human subjects review and consent to participate declarations were not applicable. This is an observational study and a clinical trial number is not applicable.

### Activities of daily living

Our outcomes were the presence of any ADL limitations (in cross-sectional analyses) and development of new ADL limitations (in longitudinal analyses). We used the Katz ADLs, which include bathing, dressing, toileting, feeding, and transferring from bed [10]. Participants were asked if they had “any difficulty” performing each of these tasks, with responses coded as a binary variable (0 = No, 1 = Yes). For each ADL, we consider individuals to have a limitation if they report “yes” for having “any difficulty.” A scale from 0–5 was constructed, with higher numbers indicating more ADL limitations. To look at change across waves, we considered the net increase in number of ADL limitations.

### Physical performance

We considered two physical performance measures: grip strength and gait speed. Details on assessment can be found from the study protocols of the Health and Retirement Study [29]. Briefly, grip strength was measured using a Smedley hand dynamometer. Participants were asked to squeeze the device in both dominant and non-dominant hands in a standing position, with their arm at a 90-degree angle by the side of the body. Two measurements per hand were obtained. We used each participant’s maximum grip strength value out of all valid measurements taken. Gait speed was measured as total time to walk a 98.5-inch (2.5-meter) course at normal pace. Participants were allowed to complete the course with a walking aid, if they normally used an aid. Two trials were recorded, and we used the participants’ fastest time. We converted the timed walk into gait speed in meters per second.

### Socioeconomic status

We considered three measures for SES: education, wealth, and income. Education was divided into four categories of highest educational attainment: less than high school, high school or General Educational Development (GED), some college (including associate’s degrees), and college and above. Wealth was measured as total household wealth, which was calculated as the value of all assets minus all debts. Income was total household income over the previous year, included earnings, pensions, and Social Security benefits. Wealth and income were transformed using rank-based inverse normal transformation to achieve normality.

### Covariates

We adjusted for age, sex, comorbidity count, and cognition. Comorbidities included in the comorbidity count were hypertension, diabetes, cancer, lung disease, heart disease, stroke, psychiatric problems, and arthritis. Cognition comprised performance in immediate and delayed word recall, orientation, object naming, serial 7 subtraction, counting backward from 20, and naming the current President and Vice President of the United States of America and was scored out of a maximum of 35 points [30]. Furthermore, regressions for grip strength included measured body mass index (BMI) and regressions for gait speed included measured height as a covariate. Continuous variables were not discretized.

### Statistical analysis

We analyzed complete-case data both cross-sectionally in 2016 and longitudinally looking at change from 2016 to 2018. Cohort characteristics were analyzed using simple descriptive statistics, including mean, median, and frequency.

To analyze the presence of ADL limitations in cross-sectional analyses or development of new ADL limitations in longitudinal analyses, we used logistic regression. We first ran regressions with either the physical performance or SES variable, examining the independent effect of each predictor. Then, we incorporated both physical performance and SES variables in the regression to understand their main effects. Then, we analyzed effect modification separately for each measure of SES with each measure of performance. For doing so, we conducted regressions with physical performance, SES, and their corresponding interaction term (the product of the two variables).

We conducted two sensitivity analyses. First, we extended the follow-up for our longitudinal analysis to 2020 from 2018, continuing to use logistic regression. Second, we ran negative binomial regressions of the count of prevalent or new ADL limitations.

Analysis was completed using R version 4.2.2.

Study funders had no role in study design, data collection and analysis, decision to publish, or preparation of the manuscript.

## Results

### Cohort characteristics

The samples for the cross-sectional grip strength and gait speed analyses comprised 4,825 and 3,401 individuals respectively, while those for longitudinal analyses were 3,995 and 2,860 respectively. Table 1 shows baseline characteristics of individuals in the analyses of grip strength and gait speed. The cross-sectional as well as longitudinal analyses cohorts for grip strength and gait speed were 57% and 59% female, respectively. Mean (SD) ages, in years, were 69.2 (11.7) and 75.4 (7.2) for the respective cross-sectional grip strength and gait speed cohorts, and were similarly 69.0 (11.4) and 75.1 (6.9) in the longitudinal cohorts. Approximately 15% of individuals in the cross-sectional cohorts had at least one ADL limitation and only 6% had more than one. These dropped to around 13% and 5% in the longitudinal cohorts. Difficulty dressing was the most common ADL limitation in all cohorts.

**Table 1:** Descriptive statistics of 2016 cross-sectional and 2016-2018 longitudinal cohorts, separated into grip strength and gait speed groups.

Variable	2016 Cross Sectional Cohort		2016-2018 Longitudinal Cohort	
	Grip Strength	Gait Speed	Grip Strength	Gait Speed
	n=4825	n=3401	n=3995	n=2860
**Age,Mean (SD)**	69.2 (11.7)	75.4 (7.2)	69.0 (11.4)	75.1 (6.9)
**Female, N (%)**	2733 (56.64%)	2007 (59.01%)	2287 (57.25%)	1691 (59.13)
**Education, N (%)**				
Less Than HS	811 (16.81%)	564 (16.58%)	621 (15.54%)	439 (15.35)
High School/GED	1603 (33.22%)	1193 (35.08%)	1323 (33.12%)	999 (34.93)
Some College	1192 (24.70%)	799 (23.49%)	980 (24.53%)	667 (23.32)
College & Above	1219 (25.26%)	845 (24.85%)	1071 (26.81%)	755 (26.40)
				
**Wealth, $10,000, Median (IQR)**	15.0 (2.2-49.5)	21.2 (5.6-62.8)	16.2 (2.9-53.2)	22.6 (6.2-65.4)
				
**Income, $10,000, Median (IQR)**	4.0 (2.1-8.1)	3.9 (2.2-7.2)	4.3 (2.2-8.3)	4.1 (2.2-7.5)
				
**# of ADL Limitations, N (%)**				
0	4125 (85.49%)	2900 (85.27%)	3469 (86.83%)	2488 (86.99%)
1	405 (8.39%)	305 (8.97%)	314 (7.86%)	234 (8.18%)
2	160 (3.32%)	111 (3.26%)	117 (2.93%)	80 (2.80%)
3	76 (1.58%)	48 (1.41%)	62 (1.55%)	35 (1.22%)
4	40 (0.83%)	26 (0.76%)	33 (0.83%)	23 (0.80%)
5	19 (0.39%)	11 (0.32%)	0 (0.00%)	0 (0.00%)
				
**Difficulty Toileting, N (%)**	223 (4.62%)	157 (4.62%)	155 (3.88%)	111 (3.88%)
**Difficulty Bathing, N (%)**	242 (5.02%)	172 (5.06%)	165 (4.13%)	110 (3.85%)
**Difficulty Eating, N (%)**	102 (2.11%)	76 (2.23%)	66 (1.65%)	46 (1.61%)
**Difficulty Dressing, N (%)**	417 (8.64%)	298 (8.76%)	317 (7.93%)	227 (7.94%)
**Difficulty Transferring from Bed, N (%)**	224 (4.64%)	127 (3.73%)	163 (4.08%)	97 (3.39%)

For longitudinal cohorts, ADL limitations are reported as number of new limitations developed over two years. IQR = interquartile range; SD = standard deviation.

### Grip strength analysis

**Fig 1:**
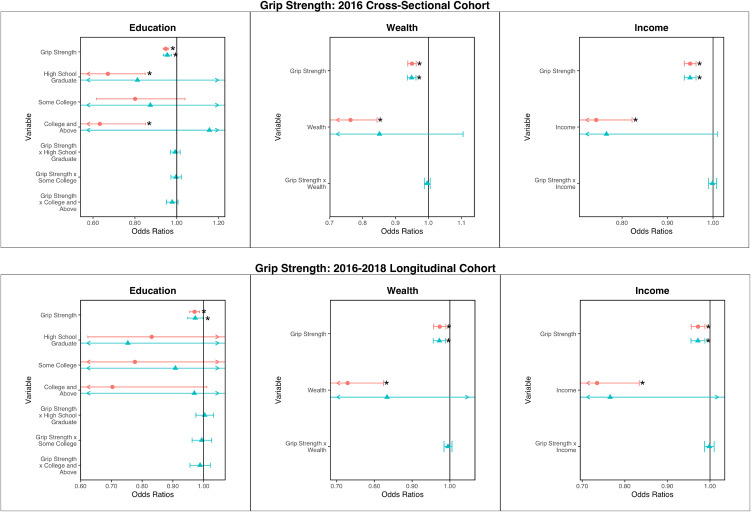
Regression results for cross-sectional associations between grip strength, socioeconomic status, and ADL limitation, presented as odds ratios with 95% confidence intervals. Red circles are for main effects-only regressions. Blue triangles are for regressions with interactions. Asterisks (*) note statistically significant associations (p < 0.05).

Fig 1 and S1 Table present the results from logistic regressions of ADL limitation on grip strength for both cross-sectional and longitudinal analyses. In the cross-sectional analysis without SES covariates, a 1-kg increase in grip strength was associated with a lower odds of having any ADL limitation (Odds Ratio [OR] (95% Confidence Interval) [95% CI]: 0.95 (0.93–0.96)). With respect to SES measures, higher levels of education were also associated with reduced odds of ADL limitation compared with less than a high school education, with ORs (95% CI) of 0.67 (0.53–0.84) for high school/GED, 0.80 (0.62–1.03) for associate’s/some college, and 0.62 (0.46–0.83) for college or above. Wealth and income had respective ORs of 0.74 (0.67–0.82) and 0.73 (0.66–0.80).

In the longitudinal analysis, the OR for grip strength was 0.97 (0.95–0.99). Compared with less than a high school education, only “College and Above” was associated with lower odds of developing a new limitation with an OR of 0.69 (0.48–0.99). Wealth and income remained significantly associated with ORs of 0.72 (0.64–0.81) and 0.73 (0.64–0.83).

In regressions including both grip strength and SES measures without interactions, greater grip strength was consistently associated with lower odds of a new ADL limitation in all cases. Income, wealth, and levels of education of high school/GED and college or above were also associated with lower odds of ADL limitation in the cross-sectional analysis. Longitudinally, only income and wealth were associated with reduced odds. Interaction terms between grip strength and SES were not statistically significant in both cross-sectional and longitudinal analyses.

### Gait speed analysis

**Fig 2:**
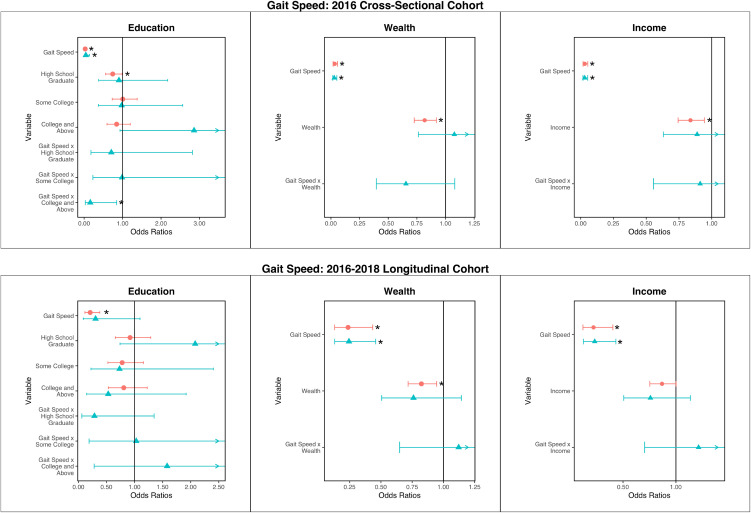
Regression results for cross-sectional associations between gait speed, socioeconomic status, and ADL limitation, presented as odds ratios with 95% confidence intervals. Red circles are for main effects-only regressions. Blue triangles are for regressions with interactions. Asterisks (*) note statistically significant associations (p < 0.05).

Fig 2 and S2 Table present the results from logistic regressions with gait speed. In cross-sectional analysis without SES covariates, a 1-meter per second increase in gait speed was linked with a reduced odds of having any ADL limitation (0.03 (0.02–0.05)). Compared with an education less than high school, having an education of GED/high school graduate was associated with lower odds of ADL limitation, with an OR of 0.69 (0.52–0.91). Wealth and income had similar associations, having ORs of 0.72 (0.64–0.81) and 0.76 (0.68–0.85), respectively.

In longitudinal analysis, the odds ratio for gait speed was 0.20 (0.11–0.37). Greater levels of education were not significantly associated with incident ADL limitations. Wealth and income did have statistically significant associations, with ORs of 0.78 (0.68–0.89) and 0.83 (0.72–0.95).

In regressions of both gait speed and SES measures without interaction terms, higher gait speed remained associated with lower odds of having an ADL limitation in all cases. In the cross-sectional analysis, GED/high school graduate education had an OR of 0.74 (0.56–0.99) of having an ADL limitation. Wealth and income were statistically significant, with ORs of 0.82 (0.73–0.92) and 0.84 (0.74–0.94) respectively. Longitudinally, only wealth was associated with lower odds of developing a new ADL limitation, with an OR of 0.82 (0.72–0.95). On including interactions between gait speed and SES, only the interaction term of “College and Above” and gait speed had a statistically significant association with ADL limitations in the cross-sectional analysis.

### Sensitivity analyses

S1 Fig and S3 Table present logistic regressions with grip strength and gait speed with follow-up extended to four years. For logistic regressions with physical performance and SES without their interaction term, the main effects of SES decreased in magnitude. Further, the only statistically significant interaction term was between gait speed and income (OR 0.56 (0.32–0.99)).

S2 and S3 Fig and S4 and S5 Tables present negative binomial regressions of the count of ADL limitations for the cross-sectional and longitudinal cohorts. In regressions with either grip strength or gait speed and SES, physical performance measures were associated with lower expected count of ADL limitation. Only several SES variables were significant, varying based on type of cohort (cross-sectional versus longitudinal) and physical performance measure used in the regression. Interactions between wealth and grip strength were found in all of these analyses (mean ratio 2016 cross-sectional: 0.99 (0.98–1.00); 2016−18 longitudinal 0.99 (0.98–1.00); 2016−20 longitudinal: 0.99 (0.98–1.00)). In the gait speed analyses, interactions with college and above education in the 2016 cross-sectional analysis (0.20 (0.05–0.83)) and with high school graduate in the 2016−20 longitudinal analysis (0.17 (0.03–0.83)) were also statistically significant.

## Discussion

In this study, we considered the relationship between physical performance, measured by grip strength and gait speed, SES, and ADL limitation. While we found consistent inverse relationships between physical performance and ADL limitations, we did not find evidence that SES was an effect modifier of these relationships. In particular, there were no interactions between physical performance and SES that were consistently observed across sensitivity analyses. Of the statistically significant interactions that were observed, such as those with education in the cross-sectional cohorts or with wealth in negative binomial regressions with gait speed, their coefficients were very close to the null and are unlikely to remain so after correction for multiple testing.

Two of the three SES measures we considered were about financial resources, which could be used to purchase home modifications or assistive devices to reduce the performance status needed to complete an ADL activity. It is possible that there was similar access to such devices and modifications across levels of wealth or income as many of our study subjects would have had Medicare coverage, which pays for some assistive devices. While these modifications and devices enable older adults maintain independence, it is also possible that they do not do so by making up for these performance measures [31,32]. In addition, populations facing extreme marginalization where this interaction may arise, such as people experiencing homelessness or undocumented immigrants, may be under-represented or too few in number in the survey. While this study does not support a model of interaction between SES measures and physical performance, it is possible that physical performance is a mediator of the association between SES and ADL limitations.

Our work relates to other studies investigating the relationship between disability and the environment. A single-arm study of 67 older adults who received home modifications showed that function, but not physical performance, improved [25]. Another study of American and European older adults found that the association between physical performance and disability was sometimes stronger in countries with greater GDP *per capita* and health service coverage [33]. Another study of Chinese older adults found that the built and neighborhood social environment mediated the relationship between SES and ADL limitation [34].

Our study has benefits from drawing on a large, representative, community-dwelling cohort with longitudinal follow-up. However, we are limited by power as only a minority of HRS participants have ADL limitations and interactions may require particularly large sample sizes to detect. While grip strength and gait speed are two well-studied performance measures, it is possible that measures focused on balance, flexibility, or power may yield different results. It is also possible that longer follow-up – beyond four years – is needed to see an interaction effect, as ADL limitations are more likely to develop with age [35]. ADL limitations were each assessed as a binary variable in logistic regressions, which may reduce some sensitivity of the outcome to the predictor variables.

## Conclusions

We find that the relationship between physical performance and ADL limitations is not altered by SES. The causal mechanisms that run from physical performance to ADL limitation, and how best to intervene on them, is still unknown. Future work could look at settings where access to assistive devices may be more limited. It is also important to consider that, rather than SES being an effect modifier of physical performance, physical performance may be one of the downstream mechanisms through which SES affects ADL limitations. Finally, other measures or aspects of physical performance may be important to consider. These may help identify new opportunities to help older adults remain independent.

## Supporting information

S1 Fig Logistic regression results for grip strength and gait speed in the 2016–2020 longitudinal cohorts, presented as odds ratios with 95% confidence intervals. The first regression consists of incident ADL limitation, regressed on physical performance and SES variables. The second regression consists of ADL limitation regressed on physical performance, SES, and their interaction term. Asterisks (*) represent statistical significance (p < 0.05).
(TIFF)

S2 Fig Negative binomial regression results for grip strength, presented as odds ratios with 95% confidence intervals. The first regression consists of ADL limitation (prevalent for the cross-sectional analyses, and incident for the longitudinal analyses) regressed on grip strength and SES variables. The second regression consists of ADL limitation regressed on grip strength, SES, and their interaction term. Asterisks (*) represent statistical significance (p < 0.05).
(TIFF)

S3 Fig Negative binomial regression results for gait speed, presented as odds ratios with 95% confidence intervals. The first regression consists of ADL limitation (prevalent for the cross-sectional analyses, and incident for the longitudinal analyses) regressed on gait speed and SES variables. The second regression consists of ADL limitation regressed on gait speed, SES, and their interaction term. Asterisks (*) represent statistical significance (p < 0.05).
(TIFF)

S1 Table Logistic regression results for grip strength, presented as odds ratios with 95% confidence intervals. Model 1 represents regressions with either grip strength or SES variable as predictor. Model 2 represents regressions with both grip strength and SES variable as predictors. Model 3 represents regressions with grip strength, SES, and their interaction term as predictors.
(XLSX)

S2 Table Logistic regression results for gait speed, presented as odds ratios with 95% confidence intervals. Model 1 represents regressions with either gait speed or SES variable as predictor. Model 2 represents regressions with both for gait speed and SES variable as predictors. Model 3 represents regressions with for gait speed, SES, and their interaction term as predictors.
(XLSX)

S3 Table Logistic regression results for gait speed and grip strength using follow-up for ADL change from 2016–2020, presented as odds ratios with 95% confidence intervals. Model 1 represents regressions with either gait speed or SES variable as predictor. Model 2 represents regressions with both for gait speed and SES variable as predictors. Model 3 represents regressions with for gait speed, SES, and their interaction term as predictors.
(XLSX)

S4 Table Negative Binomial regression results for grip strength using follow-up for ADL limitations or change, presented as mean ratios with 95% confidence intervals. Model 1 represents regressions with either gait speed or SES variable as predictor. Model 2 represents regressions with both for gait speed and SES variable as predictors. Model 3 represents regressions with for gait speed, SES, and their interaction term as predictors.
(XLSX)

S5 Table Negative Binomial regression results for gait speed using follow-up for ADL limitations or change, presented as mean ratios with 95% confidence intervals. Model 1 represents regressions with either gait speed or SES variable as predictor. Model 2 represents regressions with both for gait speed and SES variable as predictors. Model 3 represents regressions with for gait speed, SES, and their interaction term as predictors.
(XLSX)
