# Supplementary figures and images for: Socioeconomic status and the relationship of physical performance with activities of daily living among US older adults
Source: PLoS One. 2026 May 20;21(5):e0347918. doi: 10.1371/journal.pone.0347918 (PMC13189311; doi:10.1371/journal.pone.0347918)

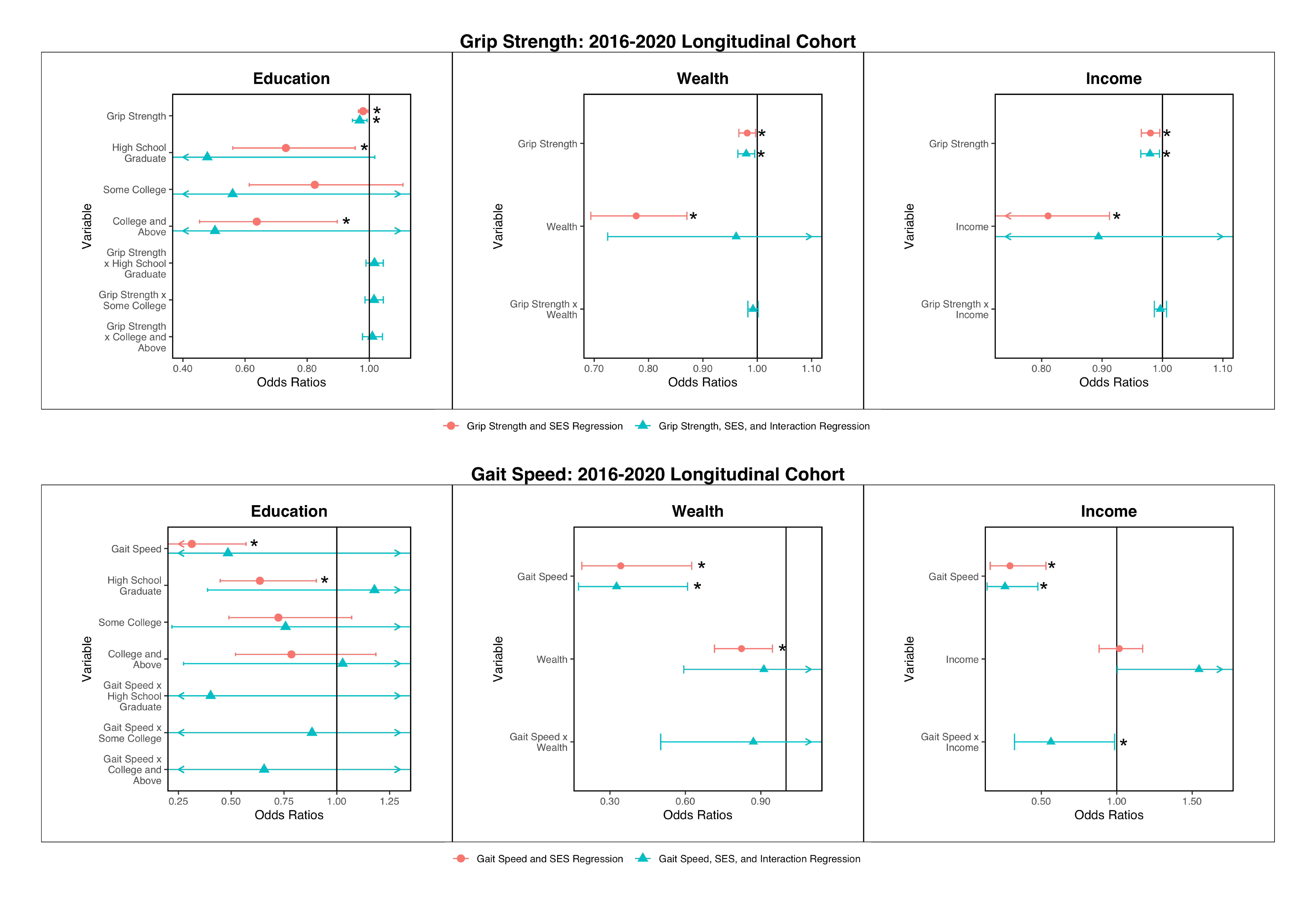

Supplement: S1 Fig — The first regression consists of incident ADL limitation, regressed on physical performance and SES variables. The second regression consists of ADL limitation regressed on physical performance, SES, and their interaction term. Asterisks (*) represent statistical significance (p < 0.05). (TIFF) [file pone.0347918.s001.tiff]
